# Postoperative Hepatic Dysfunction After Frozen Elephant Trunk for Type A Aortic Dissection

**DOI:** 10.3389/fcvm.2021.739606

**Published:** 2021-11-22

**Authors:** Shenghua Liang, Yanxiang Liu, Bowen Zhang, Yaojun Dun, Hongwei Guo, Xiangyang Qian, Xiaogang Sun

**Affiliations:** Department of Cardiovascular Surgery, The National Center for Cardiovascular Diseases, Fuwai Hospital, Chinese Academy of Medical Sciences and the Peking Union Medical College, Beijing, China

**Keywords:** aortic dissection, frozen elephant trunk, hepatic dysfunction, malperfusion, risk factors

## Abstract

**Background:** This study was aimed to investigate the incidence, risk factors, and outcomes of patients with postoperative hepatic dysfunction (PHD) after frozen elephant trunk (FET) for type A aortic dissection (TAAD).

**Method:** A retrospective study was performed with 492 patients who underwent FET for TAAD between 2015 and 2019. Independent risk factors for PHD were determined by multivariate mixed-effect logistic analysis with surgeon-specific factor as a random effect.

**Results:** The incidence of PHD was 25.4% (*n* = 125) in our cohort. Patients with PHD presented higher early mortality (10.4 vs. 1.1%, *p* < 0.001), rates of acute kidney injury (42.4 vs. 12.8%, *p* < 0.001), and newly required dialysis (23.2 vs. 3.0%, *p* < 0.001) compared with those without PHD. Moreover, with the median follow-up period of 41.3 months, the survival curve was worse in patients with PHD compared with no PHD group (log-rank *p* < 0.001), whereas it was similar after excluding patients who died within 30 days (log-rank *p* = 0.761). Multivariable analyses suggested that PHD was predicted by preoperative aspartate transferase [odds ratio (OR), 1.057; 95% confidence intervals (CI), 1.036–1.079; *p* < 0.001], celiac trunk malperfusion (OR, 3.121; 95% CI, 1.008–9.662; *p* = 0.048), and cardiopulmonary bypass time (OR, 1.014; 95% CI, 1.005–1.023; *p* = 0.003). Retrograde perfusion (OR, 0.474; 95% CI, 0.268–0.837; *p* = 0.010) was associated with a reduced risk of PHD. Celiac trunk malperfusion was an independent predictor for PHD but not associated with early mortality and midterm survival.

**Conclusions:** PHD was associated with increased early mortality and morbidity, but not with late death in midterm survival. PHD was predicted by preoperative aspartate transferase, celiac trunk malperfusion, and cardiopulmonary bypass (CPB) time, and retrograde perfusion was associated with a reduced risk of PHD.

## Introduction

Type A aortic dissection (TAAD) remains a great challenge to cardiovascular surgeons, which is a life-threatening condition with the reported 30-day mortality of 15–30% (1, 2). Total arch replacement with frozen elephant trunk (FET) can prevent the residual downstream dissection and simplify the second-stage procedure with an ideal landing zone for further endovascular operation, which is well-recommended for the treatment of aortic dissection (3, 4). Despite contemporary advances in surgical techniques, the high incidence of early morbidity still raised concerns over the risks of visceral malperfusion, and postoperative hepatic dysfunction (PHD) has been suggested to be associated with increased mortality (5, 6). The incidence and clinical outcomes of PHD after aortic dissection repair varied in terms of different definitions and patients recruited, which is hardily achieved to analyze with limited cases available. Besides, the TAAD can be complicated by extensive tear and branch artery malperfusion, such as celiac trunk malperfusion, and there have been few studies focusing on its effects after FET procedure. The goal of this study was to determine the effects of PHD on early and midterm outcomes, and evaluate the association between preoperative celiac trunk malperfusion and PHD.

## Materials and Methods

The data used in the cohort were approved by the Ethics Committee of Fuwai Hospital, with individual informed consent waived. A retrospective chart review was performed with patients who underwent FET for TAAD from January 2015 to December 2019 at our institute. The exclusion criteria were as follows: (1) aortic diseases other than TAAD, such as type B aortic dissection, aortic aneurysm, ulcer, and hematoma; (2) patients with history of heart surgery, Marfan syndrome, connective tissue disease, chronic renal failure, or severe congenital heart disease; and (3) patients with preoperative critical situation, intraoperative death, or missing data of perioperative hepatic enzymes values (details in Supplementary Figure S1). After all, a total of 492 patients were available for analyses and were divided into two groups depending on the presence of PHD: 125 patients in the PHD group and 367 patients in the no PHD group.

The primary aim of the study was to compare early clinical outcomes and midterm survival between the PHD and no PHD groups, and to determine the independent effect of the variables of interest (as shown in Supplementary Table S1) on the incidence of PHD and early mortality. In addition, a subgroup analysis was performed for the effect of celiac trunk malperfusion on PHD and survival.

**Figure 1:**
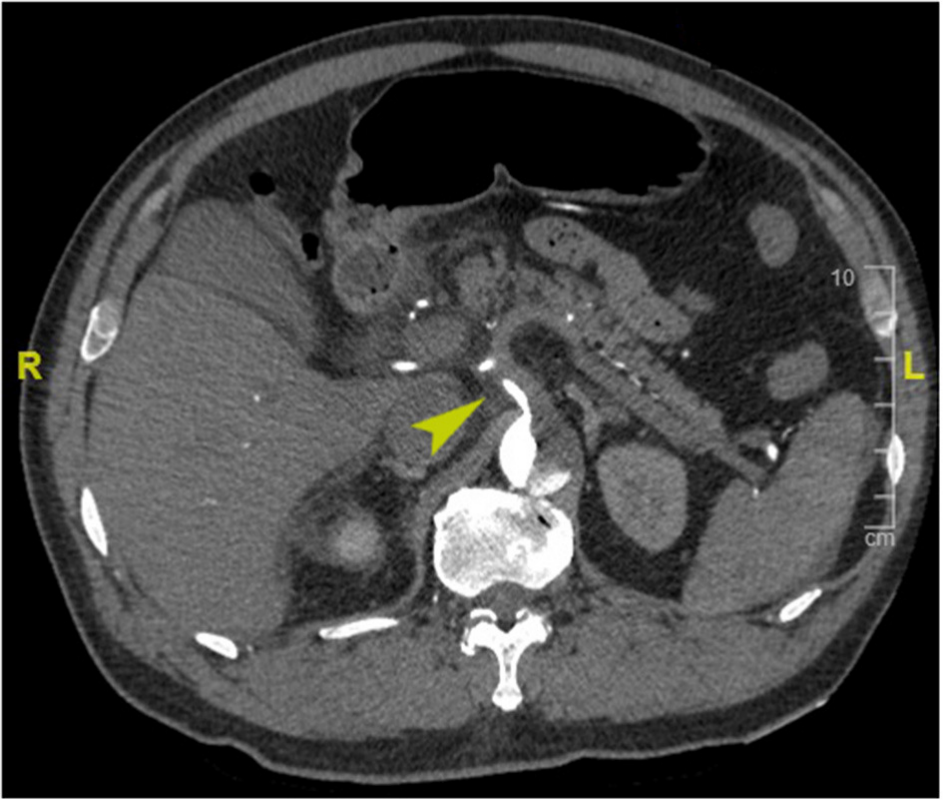
Enhanced computed tomography angiography image of a representative case of preoperative celiac trunk malperfusion (arrowhead).

PHD was defined as elevated hepatic transaminase by 1.5 times the upper range of normal within 48 h postoperatively (normal range for aspartate transferase, 0–40 IU/L; for alanine transferase, 0–50 IU/L) according to the International Aortic Arch Surgery Study Group (7). Last available preoperative hepatic transaminase before surgery was used as a baseline value. Death within 30 days of surgery was considered early mortality. Preoperative organ malperfusion was diagnosed based on clinical manifestations and laboratory tests, with CT angiography evidence confirmed (see Appendix E1 for more details). Acute kidney injury was defined as increase serum creatinine ≥1.5 times the baseline or a new requirement for dialysis within 48 h after surgery. We assessed the dissection-related anatomical information including tear extension, branch artery involvement, and branch artery malperfusion based on CT angiography. Dissection involvement referred to vessels that originated from false lumen or combined with intimal tear or malperfusion; branch artery malperfusion was defined as vessels with limited or no flow CT-enhanced signal (Figure 1). Acute dissection was referred to onset within 14 days, and emergency surgery was defined as operation performed within 24 h after hospital arrival. Retrograde perfusion was referred to performing cardiopulmonary bypass (CPB) with cannulation site of the femoral artery, including double arterial cannulation (8). We collated clinical data retrospectively from laboratory reports, radiological examination reports, and medical charts, follow-up data were obtained by telephone interview or clinic visits.

### Surgical Technique

The details of surgical approach have been described in the previous study (9). Briefly, FET procedure was performed under hypothermic circulatory arrest, and arterial cannulation for CPB was instituted at different sites according to the status of the patient (right axillary artery, femoral artery, innominate artery, and double arterial cannulation). When the nasopharyngeal temperature was dropped to 20–25°C, the aortic arch was transected between the left subclavian artery and left common carotid artery. Antegrade cerebral perfusion for brain protection through the right axillary artery was established in all patients, with a flow rate of 5–10 ml/kg/min. Aortic root repair was performed during cooling if needed. After blocking the three branch vessels of the arch, the stented FET graft (Cronus, MicroPort Endovascular Shanghai Co., Ltd., China) was implanted into the true lumen of the descending aorta with hypothermic circulatory arrest, and subsequently, we anastomosed the four-branched graft (Terumo, Vascutek Limited, Renfrewshire, UK) end-to-end with the distal aortic arch and FET graft. Restoring circulation was performed through the femoral artery (in femoral and double arterial cannulation) or the perfusion branch of the four-branched graft (in other cannulations). After reperfusion, the reconstruction of the left common carotid artery was performed first. CPB flow was returned to normal rate and rewarming was started, followed by reconstructions of the ascending aorta, left subclavian artery, and innominate artery, respectively.

### Statistical Analyses

All analyses were performed using R version 4.0.3 (R Foundation for Statistical Computing, Vienna, Austria). A two-sided *p*-value of ≤ 0.05 was considered statistically significant. The continuous variables of normal distribution were summarized as mean ± standard deviation and analyzed by one-way analysis of variance; those of non-normal distribution were expressed as median with interquartile range (IQR) and analyzed by the Kruskal–Wallis test. The Pearson's χ^2^-test or Fisher's exact test was used to compare categorical variables that were reported as frequencies with percentages.

The multivariable mixed-effect logistic regression was applied to analyze the risk factors of PHD and early mortality by glmmTMB package with surgeon-specific factor as a random effect. Between 2015 and 2020, 11 aortic surgeons performed 492 FET procedures, and 5 out of 11 surgeons completed <20 surgeries, which were calculated as one group. Clinically important variables with a value of *p* < 0.10 in univariate logistic regression were included in the multivariate logistic regression (Supplementary Table S2). The Kaplan–Meier method and log-rank test were constructed to analyze the survival rates; the multivariable Cox proportional hazard model was used to estimate the hazard ratio (HR) and 95% confidence intervals (CI) in survival function, which was applied to analyze the effect of PHD and celiac trunk malperfusion on overall survival, following variables with *p* < 0.10 on the univariate Cox analysis.

## Results

### Patient Characteristics

As shown in Table 1, the average age of patients who underwent FET was 47 ± 9 years, and 81.1% were male in our cohort. Most patients were in the presentation of acute stage (85.6%), and the dissection extension was commonly down to the iliac artery (67.7%). The percentage of acute stage (92.8 vs. 83.1%, *p* = 0.012), emergency surgery (90.4 vs. 74.4%, *p* < 0.001), preoperative liver transaminases (*p* < 0.001), and creatinine (*p* < 0.001) were higher in the PHD group compared with the no PHD group. Patients with PHD presented greater proportions of organ malperfusion (37.6 vs. 17.4%, *p* < 0.001), and the differences in ejection fraction (*p* = 0.009) and direct bilirubin (*p* = 0.003) were statistically significant but not clinically significant. In addition, significantly higher proportion of the celiac trunk (9.6 vs. 3.8%, *p* = 0.023), superior mesenteric artery (12.0 vs. 5.2%, *p* = 0.017), and iliac artery malperfusion (15.2 vs. 8.2%, *p* = 0.036) were shown in the PHD group. Otherwise, there was no significant difference identified in other preoperative characteristics between the two groups.

**Table 1:** Preoperative characteristics.

**Variables**	**All** **(*n* = 492)**	**PHD** **(*n* = 125)**	**No PHD** **(*n* = 367)**	***p*** **-value**
Age (years), mean ± SD	47 ± 9	47 ± 10	47 ± 9	0.982
Male	399 (81.1)	105 (84.0)	294 (80.1)	0.408
BMI (kg/m^2^), mean ± SD	26.4 ± 4.0	27.0 ± 4.1	26.2 ± 4.0	0.057
BSA (m^2^), mean ± SD	2.01 ± 0.20	2.04 ± 0.20	2.01 ± 0.20	0.120
Acute stage	421 (85.6)	116 (92.8)	305 (83.1)	0.012
Emergency surgery	387 (78.7)	113 (90.4)	274 (74.7)	<0.001
Hypertension	398 (80.9)	105 (84.0)	293 (79.8)	0.373
Coronary artery disease	43 (8.7)	12 (9.6)	31 (8.4)	0.833
Diabetes	16 (3.3)	3 (2.4)	13 (3.5)	0.741
COPD	5 (1.0)	0 (0.0)	5 (1.4)	0.426
Cerebrovascular accident	20 (4.1)	7 (5.6)	13 (3.5)	0.457
Smoking	216 (43.9)	57 (45.6)	159 (43.3)	0.735
NYHA grade ≥III	34 (6.9)	7 (5.6)	27 (7.4)	0.642
Ejection fraction, median (IQR)	60 (60–63)	60 (59–62)	60 (60–63)	0.009
Moderate to severe AI	52 (10.6)	15 (12.0)	37 (10.1)	0.664
**Laboratory tests, median (IQR)**				
ALT (IU/L)	21 (14–35)	24 (16–52)	20 (14–32)	0.005
AST (IU/L)	23 (17–32)	31 (20–54)	21 (17–28)	<0.001
SCr (μmol/L)	88 (73–115)	101 (80–132)	85 (70–107)	<0.001
Total bilirubin	19 (14–24)	19 (14–25)	19 (14–24)	0.931
Direct bilirubin	4 (3–6)	4 (3–7)	4 (3–5)	0.003
PT-INR	1.10 (1.06–1.18)	1.11 (1.06–1.19)	1.10 (1.06–1.17)	0.572
Albumin	39.8 (36.4–42.9)	40.6 (37.1–43.3)	39.4 (36.1–42.6)	0.054
ALT/AST ≥100 IU/L	31 (6.3)	20 (16.0)	11 (3.0)	<0.001
**Organ malperfusion**	111 (22.6)	47 (37.6)	64 (17.4)	<0.001
Myocardial	32 (6.5)	13 (10.4)	19 (5.2)	0.066
Cerebral	21 (4.3)	4 (3.2)	17 (4.6)	0.669
Visceral	28 (5.7)	20 (16.0)	8 (2.2)	<0.001
Renal	28 (5.7)	12 (9.6)	16 (4.4)	0.050
Peripheral	38 (7.7)	16 (12.8)	22 (6.0)	0.023
**Dissection extension**				
Aortic arch	54 (11.0)	12 (9.6)	42 (11.4)	0.686
Thoraco-abdominal aorta	106 (21.5)	24 (19.2)	82 (22.3)	0.540
One iliac artery	158 (32.1)	40 (32.0)	118 (32.2)	1.000
Both iliac artery	175 (35.6)	49 (39.2)	126 (34.3)	0.382
**Vessel involvement**				
Celiac trunk	195 (39.6)	51 (40.8)	144 (39.2)	0.839
Superior mesenteric artery	99 (20.1)	33 (26.4)	66 (18.0)	0.058
Renal artery	333 (67.7)	89 (71.2)	244 (66.5)	0.388
Iliac artery	333 (67.7)	89 (71.2)	244 (66.5)	0.388
**Malperfusion**				
Celiac trunk	26 (5.3)	12 (9.6)	14 (3.8)	0.023
SMA	34 (6.9)	15 (12.0)	19 (5.2)	0.017
Celiac trunk/SMA	54 (11.0)	23 (18.4)	31 (8.4)	0.004
Renal artery	60 (12.2)	21 (16.8)	39 (10.6)	0.096
Iliac artery	49 (10.0)	19 (15.2)	30 (8.2)	0.036

*Data are presented as n (%) unless otherwise stated.*

*AI, aortic insufficiency; ALT, alanine transferase; AST, aspartate transferase; BMI, body mass index; BSA, body surface area; COPD, chronic obstructive pulmonary disease; INR, international normalized ratio; IQR, interquartile range; NYHA, New York Heart Association; PHD, postoperative hepatic dysfunction; PT, Prothrombin time; SCr, serum creatinine; SD, standard deviation; SMA, superior mesenteric artery*.

### Operative Characteristics

In this cohort, 14.2% had concomitant coronary artery bypass graft procedure and retrograde perfusion was accessed in 53.9% (Table 2). Among patients undergoing FET for aortic dissection, median circulatory arrest time was 16 min (IQR, 14–19 min), and median lowest nasopharyngeal and bladder temperature on bypass were 24.1°C (IQR, 22.4–25.0°C), and 26.1°C (IQR, 24.7–27.5°C), respectively. Patients with PHD had more percentage of concomitant coronary artery bypass graft procedure (24.8 vs. 10.6%, *p* < 0.001). Longer total surgery time (439 vs. 365 min, *p* < 0.001), CPB time (183 vs. 159 min, *p* < 0.001), cross-clamping time (115 vs. 98 min, *p* < 0.001), and circulatory arrest time (17 vs. 16 min, *p* = 0.035) were found in the PHD group. Moreover, higher nasopharyngeal temperature (*p* = 0.019) was observed in patients with PHD, and the differences in intraoperative red blood cell transfusions (*p* < 0.001) and fresh-frozen plasma (*p* < 0.001) were statistically significant but not clinically significant.

**Table 2:** Intraoperative details and early outcomes.

**Variables**	**All** **(*n* = 492)**	**PHD** **(*n* = 125)**	**No PHD** **(*n* = 367)**	***p*** **-value**
**Concomitant procedure**, ***n*** **(%)**				
CABG	70 (14.2)	31 (24.8)	39 (10.6)	<0.001
Bentall procedure	119 (24.2)	34 (27.2)	85 (23.2)	0.430
David procedure	8 (1.6)	3 (2.4)	5 (1.4)	0.702
Wheat procedure	7 (1.4)	2 (1.6)	5 (1.4)	1.000
Aortic valve replacement	3 (0.6)	1 (0.8)	2 (0.5)	1.000
Ascending-femoral bypass	26 (5.3)	7 (5.6)	19 (5.2)	1.000
Retrograde perfusion, *n* (%)	265 (53.9)	63 (50.4)	202 (55.0)	0.427
**Operative duration (min), median (IQR)**				
Total surgery time	375 (317–455)	439 (355–525)	365 (311–431)	<0.001
Cardiopulmonary bypass	167 (140–202)	183 (160–253)	159 (135–193)	<0.001
Cross-clamp	100 (80–126)	115 (88–140)	98 (77–119)	<0.001
Circulatory arrest	16 (14–19)	17 (15–20)	16 (14–19)	0.035
**Intraoperative transfusion, median (IQR)**				
Red blood cells (U)	0 (0–0)	0 (0–4)	0 (0–0)	<0.001
Fresh-frozen plasma (ml)	400 (0–600)	400 (0–800)	400 (0–600)	<0.001
Platelet (U)	1 (1–1.25)	1 (1–1)	1 (1–2)	0.809
**Lowest temperature (°C), median (IQR)**				
Nasopharyngeal	24.1 (22.4–25.0)	24.5 (23.5–25.0)	24.0 (22.2–24.9)	0.019
Bladder	26.1 (24.7–27.5)	26.0 (24.8–27.8)	26.1 (24.6–27.4)	0.511
**Early adverse events**, ***n*** **(%)**				
In-hospital mortality	9 (1.8)	6 (4.8)	3 (0.8)	0.013
Early mortality	17 (3.5)	13 (10.4)	4 (1.1)	<0.001
Stroke	15 (3.0)	6 (4.8)	9 (2.5)	0.309
Paraplegia	33 (6.7)	15 (12.0)	18 (4.9)	0.011
Newly required dialysis	40 (8.1)	29 (23.2)	11 (3.0)	<0.001
Reintubation	12 (2.4)	4 (3.2)	8 (2.2)	0.762
Reoperation of bleeding	23 (4.7)	5 (4.0)	18 (4.9)	0.866
IABP support	3 (0.6)	3 (2.4)	0 (0.0)	0.021
**Laboratory tests, median (IQR)**				
ALT (IU/L)	28 (19–56)	133 (70–418)	23 (17–31)	<0.001
AST (IU/L)	57 (41–98)	215 (138–570)	47 (38–61)	<0.001
SCr (μmol/L)	148 (110–229)	232 (147–356)	134 (105–194)	<0.001
Hepatic dysfunction, *n* (%)	125 (25.4)	125 (100.0)	0 (0.0)	<0.001
Acute kidney dysfunction, *n* (%)	100 (20.3)	53 (42.4)	47 (12.8)	<0.001
Postoperative hospital stay (days), median (IQR)	12 (10–16)	13 (10–17)	12 (9–15)	0.029
ICU stay (h), median (IQR)	88 (45–136)	111 (67–201)	85 (43–118)	<0.001
Ventilation time (h), median (IQR)	21 (14–47)	35 (16–78)	20 (13–42)	<0.001

*ALT, alanine transferase; AST, aspartate transferase; CABG, coronary artery bypass graft; IABP, intra-aortic balloon pump; ICU, intensive care unit; IQR, interquartile range; PHD, postoperative hepatic dysfunction, SCr, serum creatinine*.

### Early Adverse Events

The overall early mortality in our cohort was 3.5% (*n* = 17), including 13 patients in the PHD group and 4 patients in the no PHD group (10.4 vs. 1.1%, *p* < 0.001, Table 2). The percentages of paraplegia (12.0 vs. 4.9%, *p* = 0.001), newly required dialysis (23.2 vs. 3.0%, *p* < 0.001), and intra-aortic balloon pump support (2.4 vs. 0.0%, *p* = 0.021) were higher in the PHD group. Furthermore, patients with PHD were more likely to be combined with acute kidney dysfunction vs. those without PHD (42.4 vs. 12.8%, *p* < 0.001). There was no difference in terms of stroke (*p* = 0.309), reintubation (*p* = 0.762), and reoperation of bleeding (*p* = 0.866). The postoperative in-hospital stay (*p* < 0.001), intensive care unit stay (*p* < 0.001), and ventilation time (*p* < 0.001) were longer for patients with PHD.

### Overall Survival

**Figure 2:**
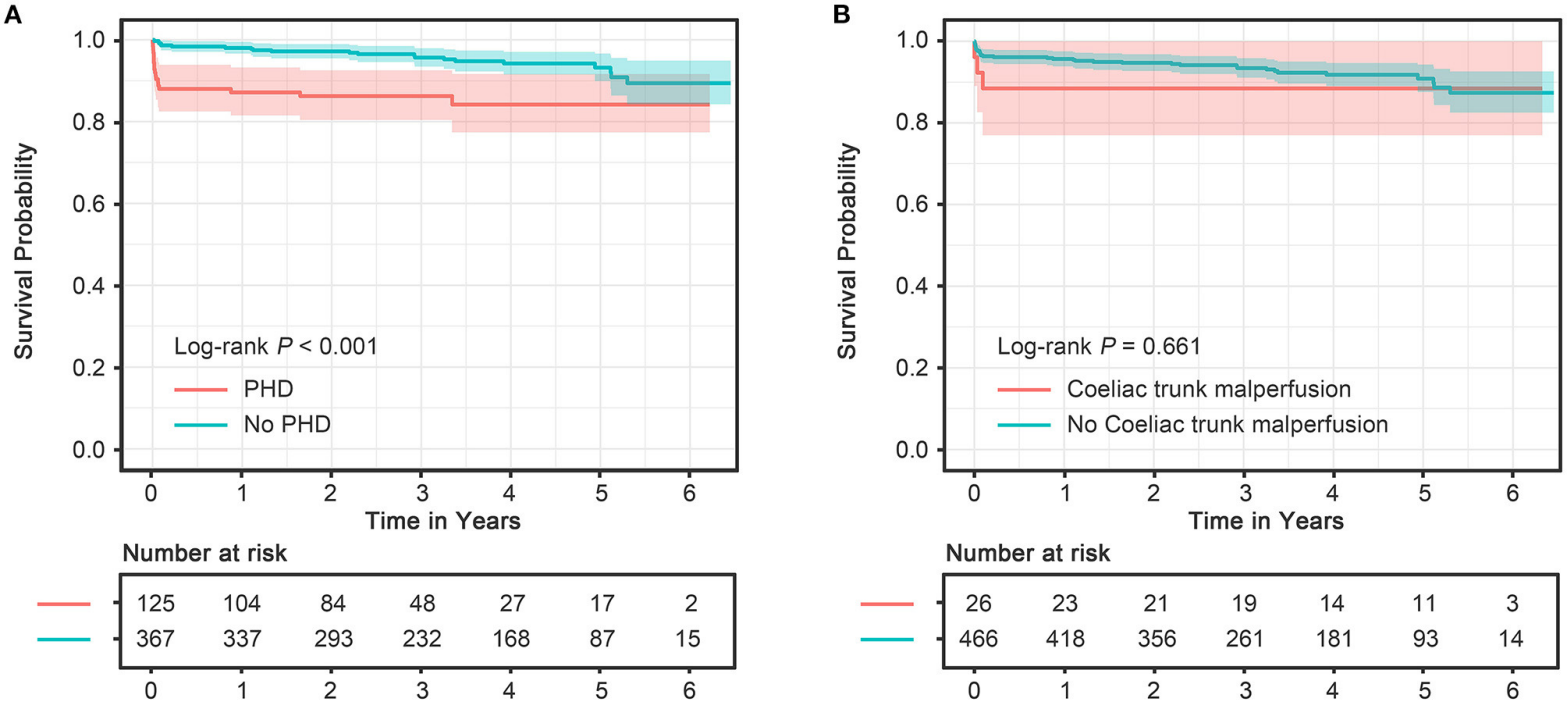
**(A)** Kaplan–Meier method estimating midterm survival of patients with and without PHD. **(B)** Kaplan–Meier method estimating midterm survival of patients with and without celiac trunk malperfusion. CI, confidence interval; HR, hazard ratio; PHD, postoperative hepatic dysfunction.

Complete follow-up was available for 453 out of 492 patients (92.1%) with a median duration of 41.3 (IQR 25.2–56.4) months, during which 18 patients died in the PHD group and 21 in the no PHD group. The Kaplan–Meier estimated cumulative survival rates at 3 and 5 years were 86.3 and 84.2%, respectively, in the PHD group, and 95.7 and 93.2%, respectively, in the no PHD group. The survival curve showed that patients with PHD were associated with worse midterm survival compared with patients without PHD (Figure 2A, log-rank *p* < 0.001), whereas similar survival curves (Supplementary Figure S2, log-rank *p* = 0.761) were observed after excluding patients that died within 30 days. The multivariate Cox proportional hazards analysis revealed that longer total surgery time predicted late death in the overall survivors (**Table 4**).

### Logistic Regression Analyses

The results of the multivariable mix-effect logistic regression are presented in Table 3 and Supplementary Table S2. Among the variables of interest, significant independent risk factors on PHD were found for preoperative aspartate transferase [Odd ratio (OR), 1.057; 95% CI, 1.036–1.079; *p* < 0.001], CPB time (OR, 1.014; 95% CI, 1.005–1.023; *p* = 0.003), celiac trunk malperfusion (OR, 3.121; 95% CI, 1.008–9.662; *p* = 0.048), early mortality for any organ malperfusion (OR, 3.571; 95% CI, 1.041–12.245; *p* = 0.043), and total surgery time (OR, 1.006; 95% CI, 1.002–1.010; *p* = 0.004). The retrograde perfusion had significantly reduced risk of PHD compared with single antegrade perfusion (OR, 0.474; 95% CI, 0.268–0.837; *p* = 0.010).

**Table 3:** The multivariable mixed effect logistic regressions.

**Variables**	**OR**	**95% CI**	***p*** **-value**
**Postoperative hepatic dysfunction**			
Preoperative AST	1.057	1.036–1.079	<0.001
Retrograde perfusion	0.474	0.268–0.837	0.010
CPB time	1.014	1.005–1.023	0.003
Celiac trunk malperfusion	3.121	1.008–9.662	0.048
**Early mortality**			
Any organ malperfusion	3.571	1.041–12.245	0.043
Celiac trunk malperfusion	3.274	0.512–20.947	0.211
Total surgery time	1.006	1.002–1.010	0.004

*The regressions were adjusted by surgeon-specific factors as a random effect.*

*AST, aspartate transferase; CI, confidence interval; CPB, cardiopulmonary bypass; OR, odds ratio*.

### Subgroup Analysis

**Figure 3:**
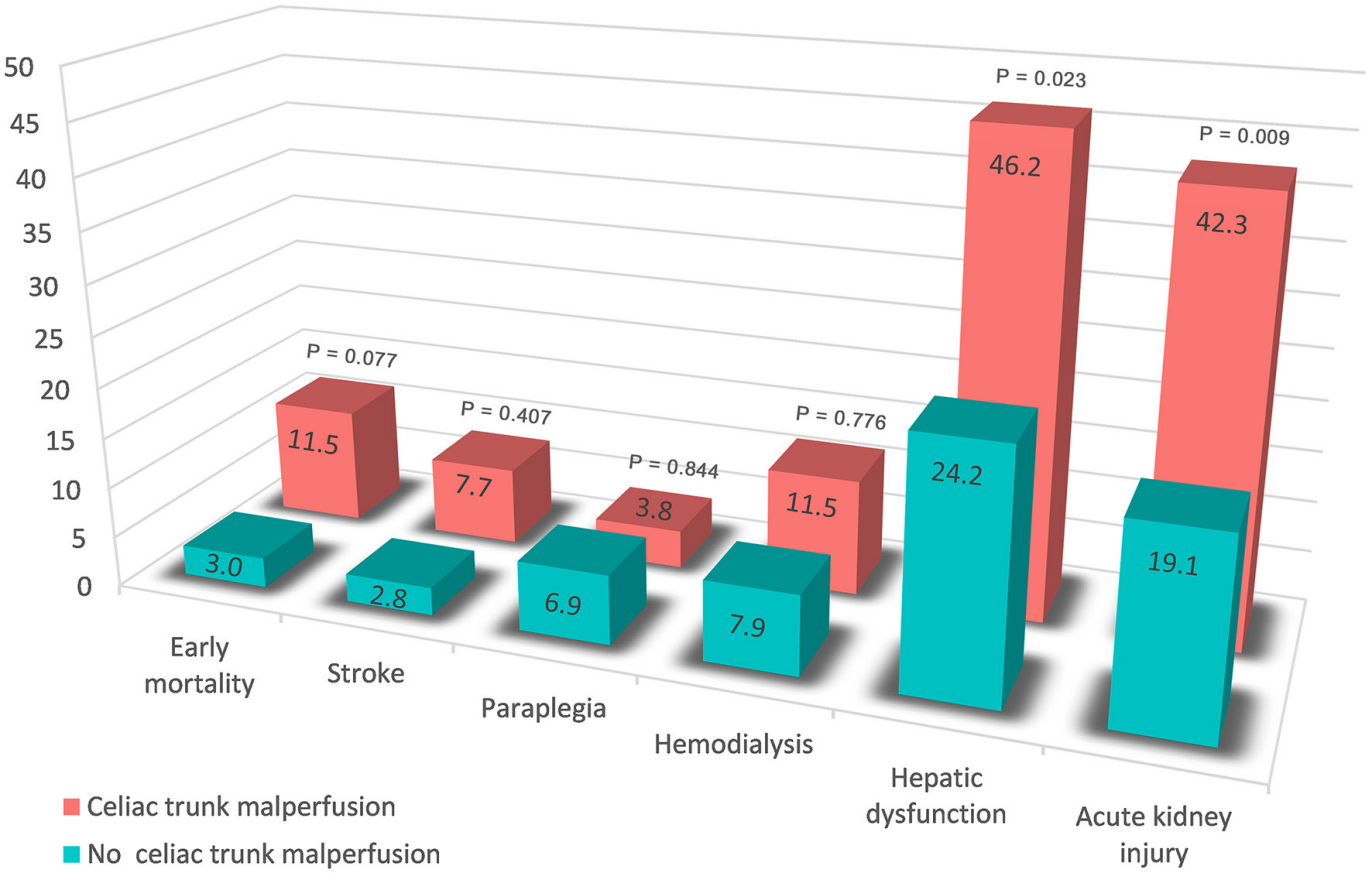
Early outcomes in the patients with and without preoperative celiac trunk malperfusion.

Of the 492 patients with aortic dissection, 26 patients (5.3%) had celiac trunk malperfusion in our cohort. Of those, the incidence of PHD (46.2 vs. 24.2%, *p* = 0.023) was much higher than those without celiac trunk malperfusion (Figure 3). Moreover, higher rates of early mortality (11.5 vs. 3.0%, *p* = 0.077) and postoperative acute kidney injury (42.3 vs. 19.1%, *p* = 0.009) were observed in celiac trunk malperfusion group. As shown in Tables 3, 4, preoperative celiac trunk malperfusion was an independent predictor for PHD but not associated with early mortality (OR, 3.274; 95% CI, 0.512–20.947; *p* = 0.211) and late death in midterm survival (HR, 1.125; 95% CI, 0.313–4.048; *p* = 0.857). No significant difference was found in the survival curve of overall survival between patients with and without celiac trunk malperfusion (Figure 2B, log-rank *p* = 0.661).

**Table 4:** The multivariable Cox proportional hazards analysis for midterm survival.

**Variables**	**HR**	**95% CI**	***p*** **-value**
Celiac trunk malperfusion	1.125	0.313–4.048	0.857
Total surgery time	1.003	1.001–1.006	0.021

*CI, confidence interval; HR, hazard ratio*.

## Discussion

In the present cohort, PHD after FET for TAAD was observed in 25.4%, and our analyses demonstrated that PHD was a severe complication associated with increased early mortality and morbidity, but not with late death in midterm survival. Preoperative celiac trunk malperfusion was an independent predictor for PHD but not associated with early mortality and midterm survival. Additionally, PHD was predicted by preoperative aspartate transferase and CPB time, and retrograde perfusion was associated with reduced risk of PHD.

Perioperative mortality in patients with aortic disease was affected by several risk factors, and PHD is one of the major factors for poor postoperative prognosis. The rate of PHD after aortic dissection repair ranged from 1.6 to 60.9% (6, 10, 11), and the differences of cohort design and definitions for hepatic dysfunction resulted in such discrepancy of incidence. While our results are in line with the findings that PHD was associated with early mortality and poor outcomes in overall survival. Also, several adverse events more frequently occurred in the PHD group. We have noticed that patients with PHD were more commonly combined with acute kidney dysfunction and the requirement of new dialysis, and similar results were reported elsewhere (6, 10, 12). The celiac trunk and renal arteries are so close that their hemodynamics might interact if a local dissection occurs. It has been reported that the preoperative renal malperfusion was an independent predictor of postoperative visceral malperfusion, and a mutual relationship might be shown between different types of malperfusion (13). Similarly, malperfusion of downstream branch arteries might suggest a narrowing true lumen combined with poor perfusion for spinal segmental arteries, and more lower extremities malperfusion and longer cross-clamp duration might also explain the higher rate of paraplegia in the PHD group.

Preoperative malperfusion of acute TAAD repair continues to be a predictor of early mortality and worse survival, and preoperative visceral malperfusion was reported to be associated with early PHD (13, 14). Clinical manifestation at presentation of organ malperfusion might lag the laboratory test and angiography imaging demonstration. Elevation of hepatic transferases or bilirubin levels reminded the presentation of preoperative liver disease, which was an independent risk factor of PHD that we found in the present cohort. On the other hand, concern about the increased risk of ischemic organ injury has been attached to dissection involvement and malperfusion in branch arteries, whereas a scarcity of data was found regarding this question. Aortic dissection often involves the arterial branches of downstream organs resulting in insufficient blood supply. Specifically, dissection involvement of the celiac trunk and mesenteric artery might cause visceral ischemia due to hypovolemia with poor collaterals, dynamic occlusion, or embolization. Also, thrombosis from progressive stenosis, such as malperfusion of the lower limb, might lead to various visceral system complications. Our results supported the above view that celiac trunk malperfusion was associated with PHD. Subgroup analysis also suggested that patients with celiac trunk malperfusion had a trend toward higher early morality rate (Figure 3) but not associated with worse midterm survival (Figure 2B), which might be related to the reperfusion time of branch vessels after surgery.

Several studies have demonstrated that CPB was associated with liver damage after aortic dissection repair (6, 10, 15). The influence of longer CPB on liver function might come from the following aspects: hypothermia, hypoxia, hemolysis, and inflammatory reaction. It has been reported that 20–25% of the blood volume in liver arteries was decreased during CPB (16), and hence, a prolonged CPB duration can induce a critical reduction of perfusion and liver damage with alterations of hepatic enzymes (17). Meanwhile, longer CPB can lead to more hemolysis, resulting in increased free hemoglobin, acceleration of the immediate release of free cyanide (18), and production of endogenous substances. The subsequent disorders of the coagulation system and inflammatory action weakens the immune response, following the possibility of multi-organ dysfunction (15), especially in the liver, kidney, lung, and other organs. This could be one of the reasons why patients with PHD were more commonly gathered with acute kidney dysfunction and the newly required dialysis in our cohort. Pacini et al. (19) have found that CPB > 180 min was independently related to liver dysfunction after aortic arch surgery, and there was a similar trend of increasing risk of PHD when CPB time exceeded the median value (167 min) in our analysis (Supplementary Figure S3).

Interestingly, retrograde perfusion predicted a lower risk of PHD compared with single antegrade perfusion. It has been reported that femoral cannulation, which offers the benefit of time-saving and rapidly instituting, is preferred for hemodynamically unstable patients during aortic disease repair (20). The protection of retrograde perfusion for PHD might be explained by short-distance, adequate, and faster perfusion for visceral arteries retrogradely through femoral access especially for those combined with true lumen narrowing of downstream (21). Otherwise, double arterial cannulation could be effective for both prevention and management of intraoperative malperfusion (8), and whether it played a role in this result needs further study.

### Limitation

The present study has certain limitations. Analyses were exploratory in nature, and this study was observational and retrospective, which was subject to selective bias. Our conclusions were limited by its execution as a single institution experience and a relatively small sample of patients. Moreover, PHD after aortic dissection repair did not have a universally accepted definition. Although some researchers used the Model for End-Stage Liver Disease score to estimate liver function, its feasibility and accuracy need to be questioned due to warfarin administration and hemodynamic changes in aortic dissection (10, 22). Besides, the dissection-related information of liver arteries was not ascertained, which might more directly derive the interplay between branch arterial malperfusion and PHD.

## Conclusions

In patients undergoing total arch replacement with FET, PHD was associated with increased early mortality and morbidity, but not with the late death in midterm survival. Higher rates of acute kidney injury and newly required dialysis occurred in the PHD group. Preoperative celiac trunk malperfusion was an independent predictor for PHD but not associated with early mortality and midterm survival. Moreover, PHD was predicted by preoperative aspartate transferase and CPB time, and retrograde perfusion was associated with a reduced risk of PHD.

## Data Availability Statement

The data analyzed in this study is subject to the following licenses/restrictions. data were not allowed to be made public according to the policy of our institute. Requests to access these datasets should be directed to the corresponding author.

## Ethics Statement

The studies involving human participants were reviewed and approved by the Ethics Committee of Fuwai Hospital. Written informed consent for participation was not required for this study in accordance with the national legislation and the institutional requirements.

## Author Contributions

SL and XS: overall responsibility. SL, YL, and BZ: conception and design. XS: obtained funding and final approval of the article. SL: statistical analysis and writing the article. HG, XQ, and XS: critical revision of the article. SL, YL, BZ, and YD: data collection. SL, BZ, and YD: analysis and interpretation. HG, XQ, and XS: project administration and resources. All authors contributed to the article and approved the submitted version.

## Funding

This work was supported by the Beijing Municipal Science and Technology Commission (Z181100001718197).

## Conflict of Interest

The authors declare that the research was conducted in the absence of any commercial or financial relationships that could be construed as a potential conflict of interest.

## Publisher's Note

All claims expressed in this article are solely those of the authors and do not necessarily represent those of their affiliated organizations, or those of the publisher, the editors and the reviewers. Any product that may be evaluated in this article, or claim that may be made by its manufacturer, is not guaranteed or endorsed by the publisher.

## Supplementary Material

Supplementary Figure 1 Consolidated Standards of Reporting Trials diagram. PCI, Percutaneous Coronary Intervention; TEVAR, Thoracic Endovascular Aortic Repair.

Supplementary Figure 2 Kaplan-Meier method estimating midterm survival of patients with and without PHD after excluding patients who died within 30 days. CI, confidence interval; HR, hazard ratio; PHD, postoperative hepatic dysfunction; SMA, superior mesenteric artery.

Supplementary Figure 3 Restricted cubic splines visualizing the relationship between cardiopulmonary bypass duration and PHD on the multivariable logistic model (*P* for non-linearity = 0.178). PHD, postoperative hepatic dysfunction.

Appendix E1 Definition of organ malperfusion.

Supplementary Table 1 Variables considered in multivariable analyses.

Supplementary Table 2 Details of the multivariable mixed effect logistic regressions.
