# Supplementary figures and images for: Postoperative Hepatic Dysfunction After Frozen Elephant Trunk for Type A Aortic Dissection
Source: Front Cardiovasc Med. 2021 Nov 22;8:739606. doi: 10.3389/fcvm.2021.739606 (PMC8645859; doi:10.3389/fcvm.2021.739606)

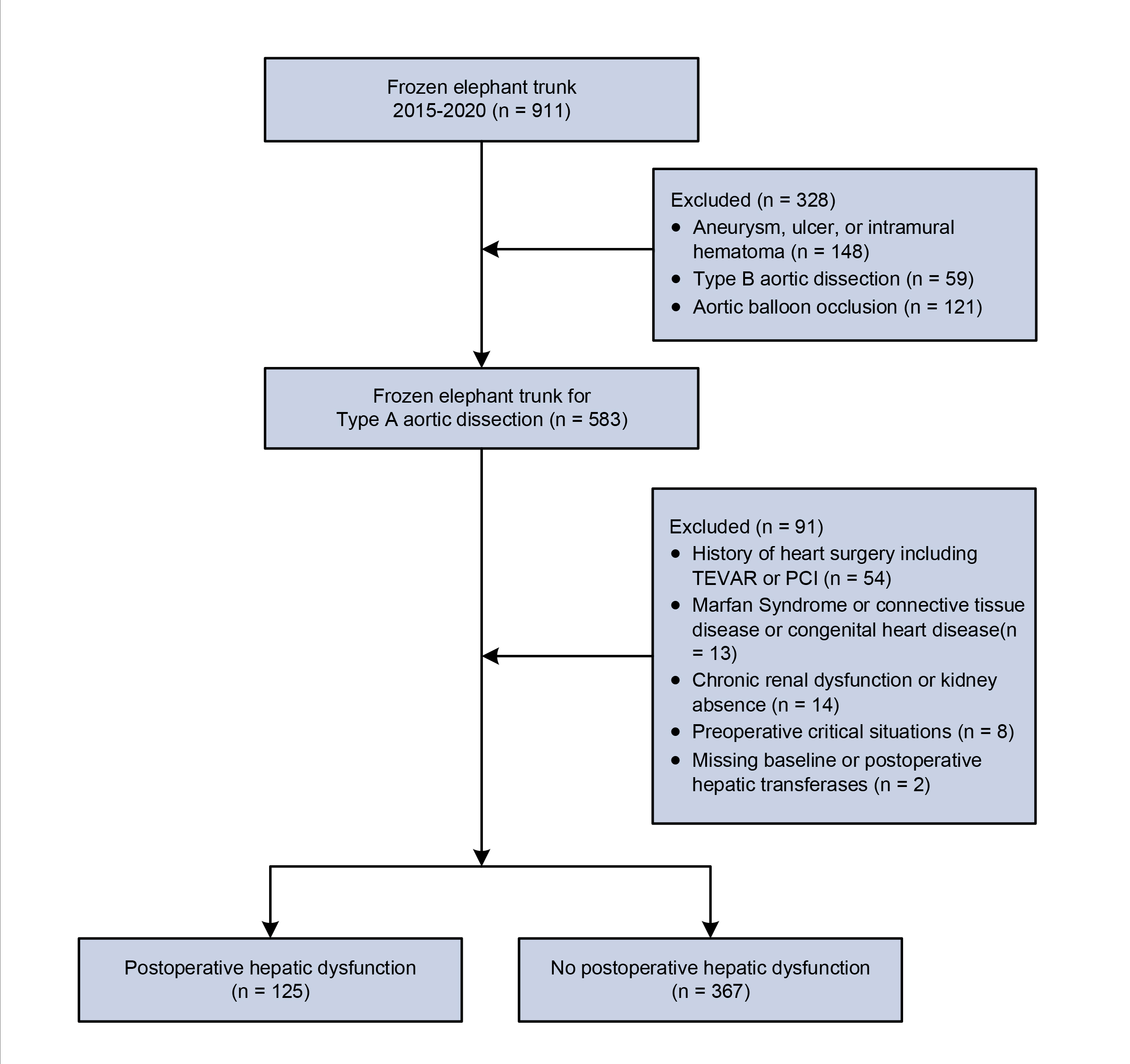

Supplement: Supplementary Figure 1 — Consolidated Standards of Reporting Trials diagram. PCI, Percutaneous Coronary Intervention; TEVAR, Thoracic Endovascular Aortic Repair. [file Image_1.JPEG]

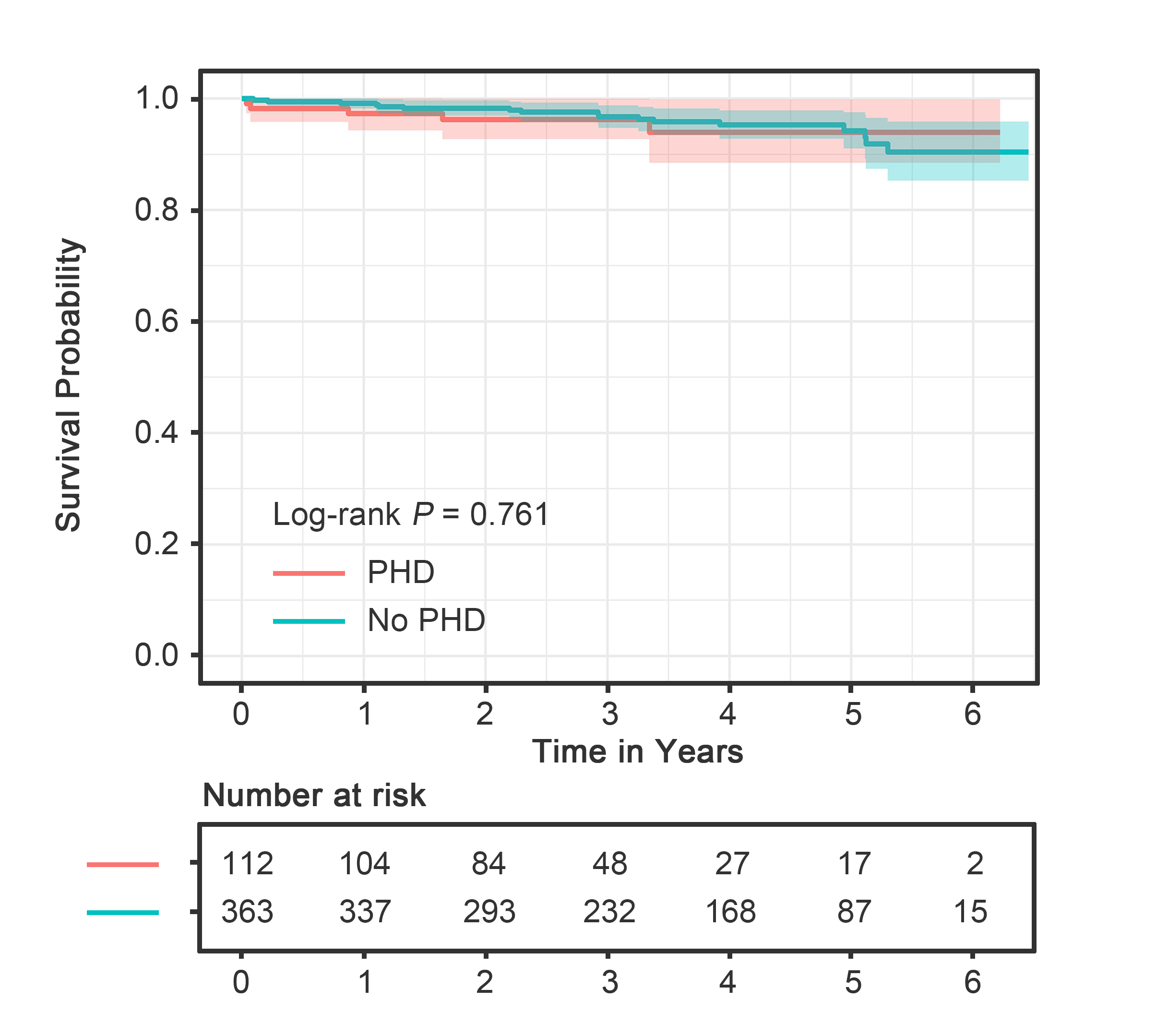

Supplement: Supplementary Figure 2 — Kaplan-Meier method estimating midterm survival of patients with and without PHD after excluding patients who died within 30 days. CI, confidence interval; HR, hazard ratio; PHD, postoperative hepatic dysfunction; SMA, superior mesenteric artery. [file Image_2.JPEG]

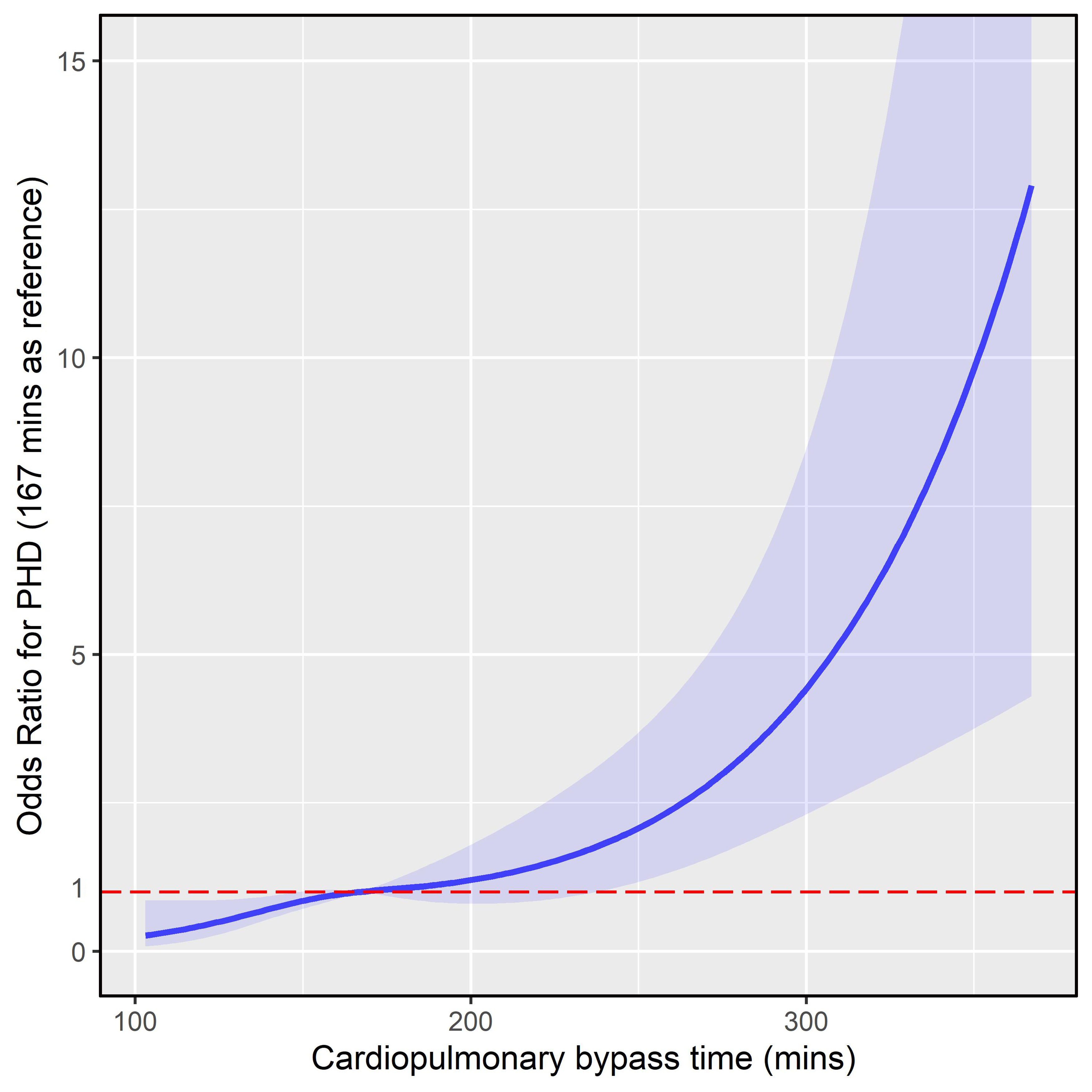

Supplement: Supplementary Figure 3 — Restricted cubic splines visualizing the relationship between cardiopulmonary bypass duration and PHD on the multivariable logistic model (P for non-linearity = 0.178). PHD, postoperative hepatic dysfunction. [file Image_3.JPEG]
