# Supplementary material for: Postoperative Hepatic Dysfunction After Frozen Elephant Trunk for Type A Aortic Dissection
Source: Front Cardiovasc Med. 2021 Nov 22;8:739606. doi: 10.3389/fcvm.2021.739606 (PMC8645859; doi:10.3389/fcvm.2021.739606)
Supplement: Appendix E1 — Definition of organ malperfusion. [file Table_1.DOCX]

**Supplementary Appendix E1.** Definition of organ malperfusion

Preoperative organ malperfusion was diagnosed based on clinical manifestations and laboratory tests, with CT angiography evidence confirmed. Myocardial malperfusion was identify on the basis of new ST-segment elevation, abnormal ventricular wall motion on echocardiogram, and significantly preoperative elevation of serum cardiac troponin I. A change in metal status, such as coma, was referred to cerebral malperfusion. Visceral malperfusion was symptoms of distention or hematochezia combined with significant liver transaminase elevation and renal malperfusion was defined as a significant increase in serum creatinine with or without decreased urine output. The lack of pulse with or without sensory or motor deficits was referred to peripheral malperfusion.

**Supplementary Table 1.** Variables considered in multivariable analyses

|  | Variables |
| --- | --- |
| Demographics | Age, sex, body mass index (kg/m^2^), body surface area (m^2^), acute dissection (within 14 days of onset) |
| Comorbidity | Hypertension, smoking, coronary heart disease, diabetes, chronic obstructive pulmonary disease, cerebrovascular accident; any organ malperfusion |
| Cardiovascular status | New York Heart Association functional grade ≥ III, ejection fraction, moderate to severe aortic insufficiency |
| Laboratory tests | Alanine transferase; aspartate transferase; alanine/aspartate transferase ≥ 100 IU/L; serum creatinine; prothrombin time - International Normalized Ratio; albumin |
| Dissection extension | Dissection extended distal to iliac artery; celiac trunk involved, superior mesenteric artery involved, renal artery involved; celiac trunk malperfusion, superior mesenteric artery malperfusion, renal artery malperfusion, iliac artery malperfusion |
| Concomitant procedures | Coronary artery bypass graft, Bentall procedure, aortic valve replacement, ascending-femoral bypass, ascending-carotid bypass, retrograde perfusion |
| Procedure | Emergency surgery (performed within 24 hours after hospital arrival), total surgery time (min), cardiopulmonary bypass time (min), circulatory arrest time (min), nasopharyngeal temperature (℃), bladder temperature (℃) |
| Random effect | Surgeon-specific factor |

**Supplementary Table 2.** Details of the multivariable mixed effect logistic regressions

| Variables | OR | 95%CI | *P* value |
| --- | --- | --- | --- |
| Postoperative hepatic dysfunction |  |  |  |
| Age | 0.986 | 0.957-1.015 | 0.346 |
| BMI | 1.057 | 0.991-1.127 | 0.089 |
| Emergency | 1.882 | 0.837-4.231 | 0.126 |
| Acute stage | 1.368 | 0.519-3.603 | 0.526 |
| Preoperative ALT | 0.985 | 0.970-1.001 | 0.072 |
| Preoperative AST | 1.057 | 1.036-1.079 | <0.001 |
| ALT/AST > 100 IU/L | 0.329 | 0.041-2.648 | 0.296 |
| Any organ malperfusion | 0.813 | 0.400-1.655 | 0.569 |
| CABG procedure | 1.652 | 0.783-3.486 | 0.188 |
| Retrograde perfusion | 0.474 | 0.268-0.837 | 0.010 |
| CPB time | 1.014 | 1.005-1.023 | 0.003 |
| Cross-clamp time | 0.990 | 0.979-1.002 | 0.096 |
| Circulatory arrest time | 0.991 | 0.934-1.050 | 0.752 |
| Nasopharyngeal temperature | 1.100 | 0.965-1.252 | 0.153 |
| Total surgery time | 1.003 | 1.000-1.005 | 0.057 |
| SMA involved | 0.947 | 0.452-1.981 | 0.885 |
| Celiac trunk malperfusion | 3.121 | 1.008-9.662 | 0.048 |
| SMA malperfusion | 2.171 | 0.723-6.522 | 0.167 |
| Renal artery malperfusion | 0.740 | 0.313-1.751 | 0.494 |
| Iliac artery malperfusion | 1.122 | 0.472-2.667 | 0.794 |
| Early mortality |  |  |  |
| Male gender | 0.452 | 0.111-1.831 | 0.266 |
| Diabetes | 1.990 | 0.178-22.206 | 0.576 |
| NYHA grade ≥ III | 2.253 | 0.478-10.615 | 0.304 |
| Preoperative ALT | 1.006 | 0.983-1.030 | 0.603 |
| Preoperative AST | 1.003 | 0.984-1.023 | 0.742 |
| Preoperative SCr | 1.000 | 0.989-1.012 | 0.939 |
| ALT/AST > 100 IU/L | 0.234 | 0.007-8.347 | 0.426 |
| Any organ malperfusion | 3.580 | 1.035-12.381 | 0.044 |
| CABG procedure | 1.360 | 0.338-5.473 | 0.665 |
| CPB time | 0.999 | 0.991-1.007 | 0.754 |
| Nasopharyngeal temperature | 1.283 | 0.940-1.751 | 0.116 |
| Total surgery time | 1.006 | 1.002-1.010 | 0.004 |
| Celiac trunk malperfusion | 3.274 | 0.512-20.947 | 0.211 |

The regressions were adjusted by surgeon-specific factors as a random effect. ALT, alanine transferase; AST, aspartate transferase; BMI, body mass index; CABG, coronary artery bypass graft; CI, confidence interval; CPB, cardiopulmonary bypass; OR, odds ratio; SMA, superior mesenteric artery.
